# Regional cerebral oxygenation measured by multichannel near-infrared spectroscopy (optical topography) in an infant supported on venoarterial extracorporeal membrane oxygenation

**DOI:** 10.1016/j.jtcvs.2011.01.026

**Published:** 2011-05

**Authors:** Maria D. Papademetriou, Ilias Tachtsidis, Murad Banaji, Martin J. Elliott, Aparna Hoskote, Clare E. Elwell

**Affiliations:** a Biomedical Optics Research Laboratory, Department of Medical Physics and Bioengineering, University College London, United Kingdom; b Cardiothoracic Unit, Great Ormond Street Hospital for Children, London, United Kingdom

In the current era of advanced cardiac surgery and extracorporeal membrane oxygenation (ECMO), there are serious limitations with inadequate neuromonitoring, misleading neuromonitoring, or both, especially in the setting of hemodilution and nonpulsatile flow.^1^ Multimodal neurological monitoring is available and advocated in certain centers.^2^ However, the implications of neurological monitoring with relevance to neurodevelopmental outcome have not been clearly delineated. As a result, there is equipoise about routine neuromonitoring, particularly with near-infrared spectroscopy (NIRS) and the relevance of data.^3^

Single- or dual-channel NIRS has been used widely in cardiac theaters.^4^ In a previous study we used dual-channel NIRS in patients undergoing ECMO to understand cerebral and peripheral tissue oxygenation.^5^ Power spectral density analysis was performed to extract vasomotion and respiratory and cardiac oscillations. To date, most NIRS studies have used optodes placed on the forehead, which monitor only a small area of the anterior cerebrum. We have developed a novel multichannel NIRS protocol for providing regional measures of cerebral oxygenation and hemodynamics for use in cardiac theaters and intensive care units. Because ECMO in the cardiac intensive care unit could be a surrogate model similar to a patient undergoing cardiac surgery during cardiopulmonary bypass, we have carried out preliminary studies on patients undergoing ECMO during manipulations in the ECMO circuit blood flows. We present our preliminary results with our first patient undergoing ECMO in which we have identified differences in regional cerebral oxygenation with changes in ECMO flows.

## Clinical Summary

**Figure 1:**
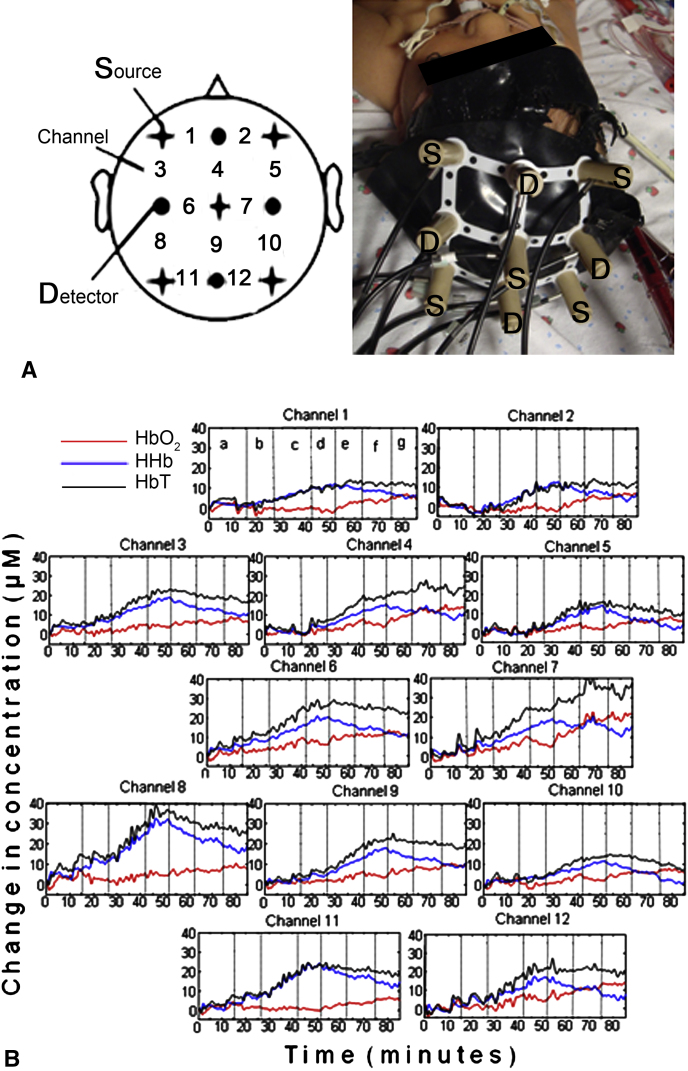
A, Schematic of the source-detector array and channel configuration. The photograph shows the UCL-designed neonatal cap in use with each source *(S)* and detector *(D)* annotated. B, Oxyhemoglobin *(HbO_2_)*, deoxyhemoglobin *(HHb)*, and total hemoglobin *(HbT)* concentration changes from 12 channels during extracorporeal membrane oxygenation circuit flow changes. The *vertical dotted lines* represent the time at which a change in flow was induced so that each section in the plots corresponds to a specific flow, as annotated on channel 1: *a*, baseline (100% flow); *b*, 90% flow; *c*, 80% flow; *d*, 70% flow; *e*, 80% flow; *f*, 90% flow; and *g*, baseline.

In this pilot study an ETG-100 optical topographic system (Hitachi Medical Ltd, Tokyo, Japan) was used to measure changes in oxyhemoglobin (HbO_2_), deoxyhemoglobin (HHb), and total hemoglobin (HbT = HbO_2_ + HHb) concentrations in a 12-day-old neonate supported on venoarterial ECMO for severe respiratory failure. ECMO circuit flow was successively decreased by 10% from initial flow every 10 to 15 minutes down to 70% of the initial flow and then gradually brought back to baseline. A novel neonatal cap was constructed to accommodate the optical sources and detectors in a 3 × 3 array (interoptode distance = 3 cm), allowing data to be collected from 12 channels (Figure 1, *A*). Multimodal data were collected synchronously with the optical data that included systemic parameters (arterial blood pressure, heart rate [HR], and arterial oxygen saturation [Spo_2_]) and ECMO circuit parameters (venous oxygen saturation [Svo_2_]).

Changes in HbO_2_, HHb, and HbT concentrations between phase I (from baseline flow [100%] to minimum flow [70%]) and phase II (from 70% flow back to baseline) were calculated from the differences in mean values over a 60-second period immediately before the change in flow. The results were analyzed with a paired *t* test (*P* < .05).

Figure 1, *B*, shows concentration changes in HbO_2_, HHb, and HbT collected from the 12 channels during changes in ECMO flows. Significant changes in HHb, HbO_2_, and HbT concentrations were seen across all channels. During phase I, a decrease in ECMO flow was associated with a substantial increase in HHb concentrations in all channels (range, 9.7 to 25.1 μmol/L). Much smaller changes were seen in HbO_2_ concentrations (range, −4.3 to 3.8 μmol/L).

**Figure 2:**
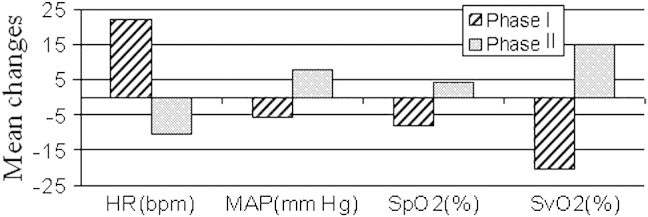
Mean changes in systemic and extracorporeal membrane oxygenation parameters during phases I and II. *HR*, Heart rate; *MAP*, mean arterial pressure; *Spo_2_*, arterial oxygen saturation; *Svo_2_*, venous oxygen saturation.

Figure 2 shows the responses of HR, mean arterial pressure, Spo_2_, and Svo_2_ during phases I and II. In this patient a decrease in flow is associated with a decrease in Svo_2_ and Spo_2_ and an increase in HR and mean arterial pressure. The effect is reversed when the flow is increased back to baseline values. Similar to hemoglobin concentrations, these systemic and ECMO parameters do not return to their baseline values by the end of the monitoring period.

## Discussion

This single-patient study showed significant changes in systemic oxygenation and cerebral hemoglobin concentrations in response to modest changes in ECMO flows. Regional variations were observed between channels 8 and 10, which potentially cover different hemispheres. Reduction in flows was associated with a decrease in Svo_2_ and Spo_2_ suggestive of a decrease in oxygen delivery. This is reflected in the NIRS data with a significant increase in HHb concentrations. The lack of a consistence decrease in HbO_2_ concentrations could be explained by a compensatory arterial dilation.

We demonstrate that multichannel optical topographic analysis can provide information on regional cerebral hemodynamics and oxygenation in patients supported by ECMO. Simultaneous measurement of systemic and cerebral HbO_2_ and HHb concentrations can help elucidate mechanisms related to the response of the brain during changes in ECMO and cardiopulmonary bypass flows. In this patient modest changes in ECMO flows appear to present a significant hemodynamic challenge to cerebral circulation. Further work is necessary to support application of this novel brain-monitoring technology in cardiac theaters and intensive care units, and we now have a protocol to further investigate regional brain oxygenation in these patients.
